# The impact of breast reduction surgery on breastfeeding: Systematic review of observational studies

**DOI:** 10.1371/journal.pone.0186591

**Published:** 2017-10-19

**Authors:** Roni Y. Kraut, Erin Brown, Christina Korownyk, Lauren S. Katz, Ben Vandermeer, Oksana Babenko, M. Shirley Gross, Sandy Campbell, G. Michael Allan

**Affiliations:** 1 Department of Family Medicine, University of Alberta, Edmonton, Canada; 2 Division of Plastic Surgery, Department of Surgery, University of British Columbia, Vancouver, Canada; 3 Winnipeg Public Library, Winnipeg, Canada; 4 Alberta Research Centre for Health Evidence, University of Alberta, Edmonton, Alberta, Canada; 5 Department of Obstetrics and Gynecology, University of Alberta, Edmonton, Alberta, Canada; 6 J.W. Scott Health Sciences Library, University of Alberta, Edmonton, Canada; Universita degli Studi di Roma La Sapienza Facolta di Medicina e Psicologia, ITALY

## Abstract

**Background:**

Almost half a million breast reduction surgeries are performed internationally each year, yet it is unclear how this type of surgery impacts breastfeeding. This is particularly important given the benefits of breastfeeding.

**Objectives:**

To determine if breast reduction surgery impacts breastfeeding success and whether different surgical techniques differentially impact breast feeding success.

**Methods:**

Databases were searched up to September 5, 2017. Studies were included if they reported the number of women successful at breastfeeding or lactation after breast reduction surgery, and if they reported either the total number of women who had children following breast reduction surgery, or the total number of women who attempted to breastfeed following surgery.

**Results:**

Of 1,212 studies, 51 studies met the inclusion criteria; they were located worldwide and had 31 distinct breast reduction techniques. The percentage of breastfeeding success among studies was highly variable. However, when analyzed by the preservation of the column of parenchyma from the nipple areola complex to the chest wall (subareolar parenchyma), a clear pattern emerged. The median breastfeeding success was 4% (interquartile range (IQR) 0–38%) for techniques with no preservation, compared to 75% (IQR 37–100%) for techniques with partial preservation and 100% (IQR 75–100%) for techniques with full preservation.

**Conclusions:**

Techniques that preserve the column of subareolar parenchyma appear to have a greater likelihood of successful breastfeeding. The preservation of the column of subareolar parenchyma should be disclosed to women prior to surgery. Guidelines on the best breast reduction techniques to be used in women of child bearing years may be advantageous to ensure women have the greatest potential for successful breastfeeding after breast reduction surgery.

## Introduction

Breastfeeding has been shown to provide substantial benefits to infant and maternal health [1,2]. The World Health Organization (WHO) recommends exclusive breastfeeding of babies/newborns up to six months of age, with continued breastfeeding along with appropriate complementary foods up to two years of age or beyond [3]. The global target is a 50% rate of exclusive breastfeeding at six months by 2025; currently the rate is at 38% [4]. Other governmental, public health, and medical agencies echo the recommendations of the WHO [1,2,5].

Breast reduction surgery is the eighth most common plastic surgery procedure performed globally, with approximately 432,000 breast reduction surgeries performed in 2015 [6]. It has been shown to improve a woman’s quality of life, including decreasing shoulder, back, and neck pain, reducing headaches, and decreasing anxiety and depression [7–9]. Breast reduction techniques have been in a continuous state of development since the early 1900s, with new techniques developed, refined, and modified by subsequent plastic surgeons [10]. This has led to many diverse breast reduction techniques [10].

While there is evidence that both breastfeeding and breast reduction surgery are beneficial, it is unknown whether breast reduction surgery impacts breastfeeding and whether any breast reduction technique differentially preserves the ability to breastfeed. A previous systematic review began to tackle these issues [11]; however, due to methodological issues, the answer still remains unclear. Therefore, our objective was to perform a systematic review to further examine these key questions. We reviewed all studies that assessed the success of breastfeeding in women after breast reduction surgery, with or without control groups.

## Methods

The standards outlined in the Preferred Reporting Items for Systematic Review and Meta-Analysis (PRISMA) were followed (S1 Table) [12].

### Search strategy

Database searches were performed by a medical librarian (S.C.) in December 23, 2014, and updated September 5, 2017, using subject headings and text words to retrieve articles related to the following concepts: breast reduction or mammoplasty and breastfeeding or lactation. Individual case reports were excluded from the searches. No other restrictions were applied. All databases were searched from their inception to September 5, 2017, without language restriction.

Databases that were searched included: OVID Medline, OVID Embase, OVID all EBM Review databases (including the Cochrane Database of Systematic Reviews), Proquest Dissertations & Theses, EBSCO CINAHL, and Scopus. Search strategies were adjusted accordingly for each database (see S1 Text which lists all search strategies). Google Scholar was also searched with “breastfeed” and "breast reduction"; the first 15 pages of results were reviewed.

References of all included studies were reviewed. Foreign language studies were translated using Google translate. No authors were contacted for further information.

### Study selection

Studies were included if they provided the number of women successful at breastfeeding or lactation after breast reduction surgery, and if they reported either the total number of women who had children following breast reduction surgery or the total number of women who attempted to breastfeed following surgery. Successful breastfeeding was based on the definition used in each reviewed study.

Two independent reviewers (R.K. and C.K.) assessed all identified studies. First, titles and abstracts were evaluated to identify possible studies for inclusion. An article was then reviewed in full if its title and/or abstract were determined to be relevant. Disagreements were resolved with consensus between the two reviewers. Percent agreement in study selection was computed.

### Data extraction

The following data were extracted from all included studies:

Study characteristics: (1) study citation: title, publication year, journal title, volume, pages, first three authors, last author, and language; (2) study location: country, state/province, and city (in cases when studies did not specify the location, the location of the authors was used as the study location); (3) definition of successful breastfeeding: number of weeks breastfeeding and exclusive or any breastfeeding. If a study did not provide a definition of breastfeeding, we considered it as “any” breastfeeding. If a study had several time points, we selected the definition closest to the WHO definition of breastfeeding success (six months), unless otherwise noted; and (4) study methods: type of study, focus of study (breastfeeding or other), sample size estimation, location of participant recruitment (plastic surgeon office, hospital or other), control group and study group drawn from the same population, outcome assessors (participant or study authors), blinding of outcome assessors, length of follow up period, statistics used, and whether confounding variables were considered.

Participant characteristics: (1) number of participants: number of women available for recruitment into the study, number of women who agreed to participate, number of women with children, and number of women who attempted breastfeeding; (2) average Body Mass Index (BMI); (3) average age at surgery; (4) percentage of women informed about the impact of breast reduction on breastfeeding prior to surgery; (5) breastfeeding supports; (6) reasons for not attempting to breastfeed; and (7) reasons for not breastfeeding successfully.

Breast reduction surgery characteristics: (1) name of breast reduction technique; (2) average tissue removed per breast in grams; (3) pedicle location, meaning the location of parenchyma left connected to the nipple areola complex; (4) preservation of column of parenchyma between the nipple areola complex and the chest wall (subareolar parenchyma), with none meaning the column of subareolar parenchyma was fully transected and there was no intact connection between the nipple areola complex and the chest wall; portion meaning part of this column was preserved intact; and entire meaning the entire column of subareolar parenchyma was preserved unaltered; and (5) width of pedicle.

Study results: (1) number of women successful at breastfeeding; (2) whether characteristics between women successful and not successful at breastfeeding were examined (e.g. BMI); (3) percent of women satisfied with the surgery; and (4) study conclusion.

Author profession was obtained through information in the study or Google search and was classified as plastic surgeon, plastic surgery affiliation, other, or unknown.

Two reviewers (R.K. and G.M.A.) developed a data extraction sheet and extracted data from the first four studies together to calibrate the data extraction between reviewers. Subsequently, two reviewers (R.K. and E.B.) independently extracted data on pedicle characteristics and two reviewers (R.K. and L.K.) independently extracted all other variables. Disagreements were resolved through consensus between the two reviewers. Percent agreement in data extraction was computed.

### Quality assessment

We used the risk of bias assessment in Sobhy et al’s systematic review as a template [13].

For studies without control groups, we used the following criteria to assess the risk of bias: adequacy of sample size, representativeness of the population, measurement, and outcome assessment. We considered sample size to be adequate if the study had 20 or more women with children, and inadequate if the study had fewer than 20 women with children. We considered a study adequate in terms of representativeness if no subsets of patients were excluded, and inadequate if a subset was excluded or it was unclear from the description provided. We deemed measurement to be adequate when the study provided a definition of breastfeeding success, and inadequate when a study did not provide a definition of breastfeeding success. We determined outcome assessment to be adequate when a study followed breastfeeding for at least six months, and inadequate when a study followed breastfeeding for less than six months. If ≥3 criteria were met, the study was deemed to have a low risk of bias. Otherwise, it was considered to have a high risk of bias.

For studies with control groups, we used the Newcastle-Ottawa scale to determine the risk of bias in selection, comparability of cohorts, and outcome assessment [14]. Studies that had four stars for selection, two stars for comparability, and three stars for ascertainment of the outcome were deemed to have a low risk of bias. Studies with two or greater stars for selection, one for comparability, and two for outcome ascertainment were considered to have a medium risk of bias. All other studies were considered to have a high risk of bias.

### Analysis

For each study, we calculated the percent of women successful at breastfeeding and used the normal scores method [15] to compute the 95% confidence interval, using the number of women with children as the denominator. We displayed these results graphically using a forest plot, but due to clinical and methodological heterogeneity across the studies, we chose not to pool the studies statistically. We considered a meta-analysis for studies with control groups, but due to the substantial heterogeneity in definitions of successful breastfeeding and in breast reduction techniques, we chose not to perform it.

Sensitivity analysis was completed for 11 variables, including (1) study characteristics: type of study, primary focus of study, profession of first author, duration of breastfeeding, and risk of bias; (2) participant characteristics: average age and surgery location; (3) surgery characteristics: average tissue removed per breast, publication year, pedicle location, and preservation of column of subareolar parenchyma. We had also wanted to include the width of the pedicle; however, too few studies provided this information.

The variables, with the exception of preservation of column of subareolar parenchyma and pedicle location, were selected a priori but their categorization (e.g., publication year cut-offs for publication year) was determined post-hoc. Subgroups were displayed graphically on a forest plot as median success with interquartile ranges (IQR).

The same analyses, breastfeeding success forest plot and sensitivity analysis, were repeated using women who attempted to breastfeed as the denominator (instead of using women with children as the denominator).

## Results

### Search strategy and study selection

**Fig 1:**
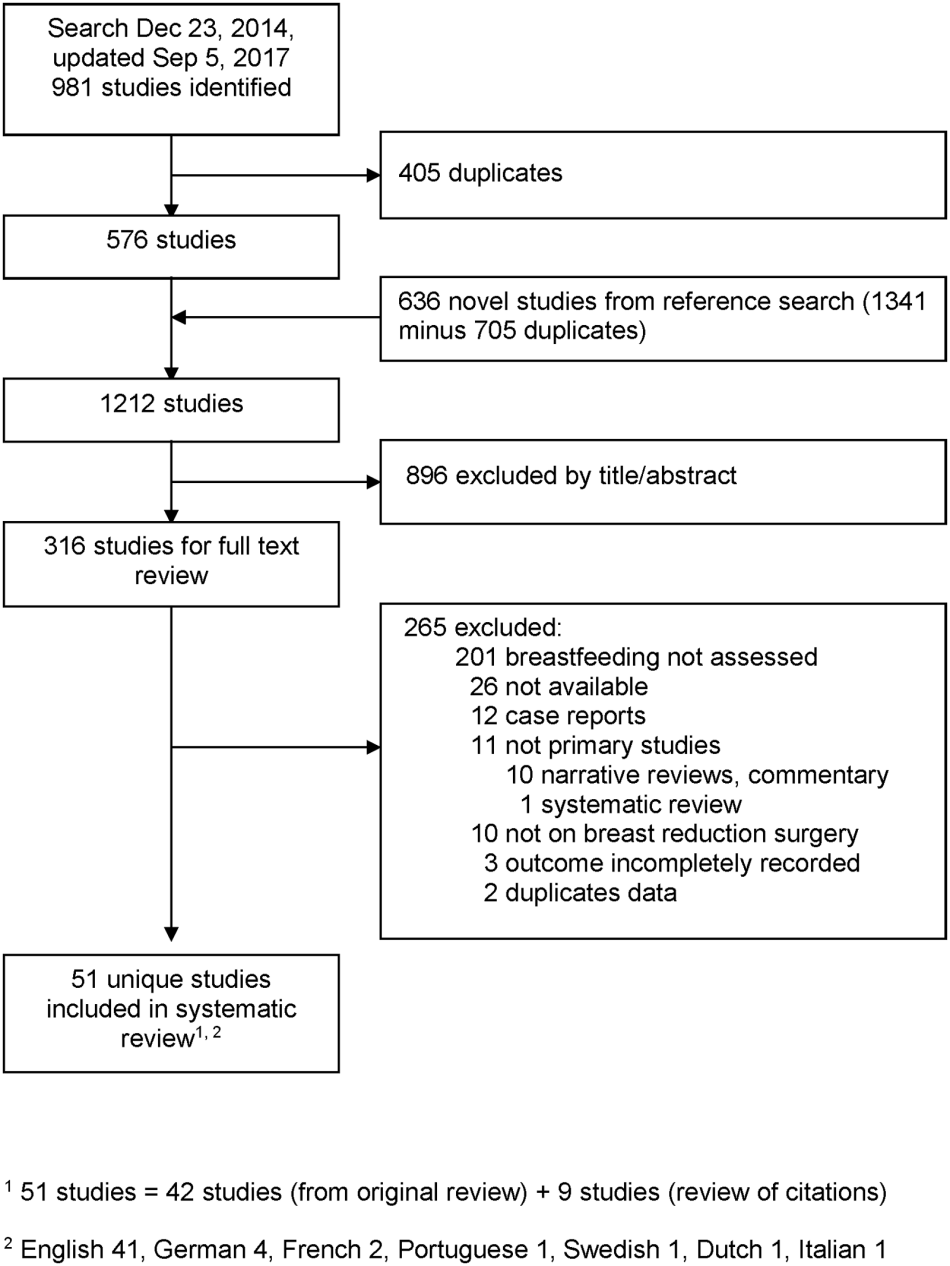
Study selection flow diagram.

Fig 1 summarizes the search results and the selection process. In total, 1,212 studies were found. Fifty-one studies [16–66] met the selection criteria; 42 from the original search [16–24, 27–34,36–41,43–50,52–55,57,61,64,65] and 9 from the citation review [25,26,35,42,51,56,62,63, 66]. Ten of the included studies were in languages other than English [23,26,35,39,55,63–66]. The agreement for study selection between the two reviewers was 89%.

### Data extraction

Characteristics of included studies are provided in Table 1 (see S2 Table for additional study details). Agreement between the two reviewers for data extraction on pedicle characteristics was 89%; the agreement for the remaining data extraction was 95%.

**Table 1:** Characteristics of included studies.

Study	Location	Pedicle location	Breast-feeding focus	Definition of breastfeeding success	Number with children	
					With reduction	Without reduction
Aboudib, 1991[16]	Brazil, Rio de Janeiro	Superior	No	Any	11	Not given
Aillet, 2002[17]	France, Rennes	Superior (77%), lateral (12%), inferior (6%), not given (3%), not given (2%)	Yes	Any	17	Not given
Akpuaka, 1998[18]	Nigeria, Enugu	Inferior	No	Any	10	Not given
Atterhem, 1998[19]	Sweden, Umea	Horizontal bipedicle (62%), lateral (27%), superior (7%), free nipple (4%)	No	Any	12	Not given
Bretteville-Jensen, 1976[20]	Denmark, Copenhagen	Vertical bipedicle	No	Any	4	Not given
Brzozowski, 2000[21]	Canada, London	Inferior	Yes	Exclusive 2 weeks	78	Not given
Buenaventura, 1996[22]	USA, Charlotte	Inferior (81%), central (16%), free nipple graft (3%)	No	Any	5	Not given
Caouette-laberge, 1992[23]	Canada, Montreal	Inferior and superior	Yes	Any 3 weeks	18	Not given
Cardenas-Camarena, 2001[24]	Mexico, Guadalajara	Superior/lateral	No	Any	Not given	Not given
Cardoso de Castro, 1978[25]	Brazil, Rio de Janeiro	Superior	No	Any	3	Not given
Cardoso de Castro, 1986[26]	Brazil, Rio de Janeiro	Superior	No	Any	6	Not given
Chen, 1997[27]	Taiwan, Kaohsiung	Superior	No	Any	Not given	Not given
Cherchel, 2007[28]	Belgium, Brussels	Superior	Yes	Exclusive 2 weeks	18	7
Chiummariello, 2008[29]	Italy, Rome	Lateral (28%), superior (27%), medial (23%), inferior (22%)	Yes	Exclusive 3 weeks	105	Not given
Copcu, 2009[30]	Turkey, Aydin	Central U shape	No	Exclusive 24 weeks	6	Not given
Cruz-Korchin, 2004[31]	Puerto Rico, San Juan	Medial	Yes	Exclusive 2 weeks	58	149
Cruz, 2007[32]	Puerto Rico, San Juan	Medial (36%), inferior (35%), superior (29%)	Yes	Exclusive 2 weeks	164	151
de Andrade, 2010[33]	Brazil, Sao Paulo	Not given	Yes	Exclusive 4 weeks	25	25
Deutinger, 1993[34]	Austria, Vienna	Vertical bipedicle (44%), horizontal bipedicle (31%), superior (25%)	Yes	Any	15	5,635
Festge, 1960[35]	Not given	Central	No	Any	Not given	Not given
Hang-Fu, 1991[36]	USA, New Brunswick	Free nipple (29%), vertical bipedicle (27%), superior (14%), modified superior (13%), inferior (11%), horizontal bipedicle (6%)	No	Any	Not given	Not given
Harris, 1992[37]	Canada, Toronto	Inferior	Yes	Any 8 weeks	20	Not given
Hefter, 2003[38]	Norway, Tromse	Lateral	Yes	Exclusive 8 weeks	13	Not given
Hintringer, 1994[39]	Austria, Linz	Superior	No	Any	18	80
Hughes, 1993[40]	USA, Washington DC	Not given	Yes	Any	23	Not given
Kakagia, 2005[41]	Greece, Alex/polis	Horizontal bipedicle (41%), inferior (37%), superior (22%)	Yes	Exclusive 3 weeks	97	Not given
Kallen, 1986[42]	Sweden, Helsingborg	Horizontal bipedicle	No	Any	3	Not given
Kappel, 1997[43]	Netherland, Zwolle	Superior (50%), central (50%)	Yes	Any	16	Not given
Lee, 2003[44]	USA, Akron	Verticle bipedicle (56%), inferior (35%), superior pedicle or free nipple graft (9%)	No	Any	Not given	Not given
Letertre, 2009[45]	France, NG	Inferior	No	Any	Not given	Not given
Lossing, 1985[46]	Sweden, Gothenburg	Superior	Yes	Any 4 weeks	22	Not given
Makki, 1998[47]	Qatar, Doha	Inferior	No	Any	36	Not given
Mandrekas, 1996[48]	Greece, Athens	Inferior	No	Any	18	Not given
Marshall, 1994[49]	Australia, Melbourne	Inferior (68%), horizontal bipedicle (23%), vertical bipedicle (5%), free nipple graft (4%)	Yes	Exclusive 12 weeks	29	347
McMahan, 1995[50]	USA, Ohio	Superior, inferior, lateral, vertical, strombeck and free nipple graft	No	Any	9	Not given
Moufarrege, 1990[51]	Canada, Montreal	Central	No	Any	54	Not given
Muller, 1974[52]	Germany, Bochum	Horizontal bipedicle	No	Any	10	Not given
Nguyen, 2013[53]	United States, Olmstead County	Not given	No	Any	72	Not given
Pers, 1986[54]	Denmark, Copenhagen	Vertical bipedicle	No	Any	77	Not given
Portincasa, 2008[55]	Italy, Foggia	Central (50%), superior (28%), inferior (22%)	No	Any	11	Not given
Ragnell, 1957[56]	Sweden, Stockholm	Inferior (81%), unclear (19%)	Yes	Any 24 weeks	27	105
Ramirez, 2002[57]	United States, Baltimore	Superior/central	No	Any	2	Not given
Sandsmark, 1992[58]	Norway, Oslo	Superomedial bipedicle (80%), inferior (12%), superior (4%), superolateral (3%), free nipple graft (1%), horizontal bipedlcle (.5%)	No	Exclusive	42	Not given
Sinno, 2013[59]	Canada, Montreal	Central	Yes	Exclusive 24 weeks	Not given	Not given
Souto, 2003[60]	Brazil, Porto Alegre	Not given	Yes	Exclusive 12 weeks	49	96
Strombeck, 1964[61]	Sweden, Stockholm	Horizontal bipedicle	Yes	Any 12 weeks	118	411
Strombeck, 1964[62]	Sweden, Stockholm	Horizontal bipedicle	No	Any 4 weeks	Not given	Not given
Strombeck, 1981[63]	Sweden, Stockholm	Horizontal bipedicle	No	Any 24 weeks	30	Not given
Tairych, 2000[64]	Austria, Vienna	Inferior (36%), central (28%), superior (18%), vertical bipedicle (10%), free nipple (8%)	Yes	Any 24 weeks	28	Not given
Witte, 2004[65]	Netherlands, Leeuwarden	Superior, horizontal bipedicle	Yes	Any 1 week	215	Not given
Wuringer, 1999[66]	Austria, Vienna	Central	No	Any	2	Not given

The selected studies were published between 1957 and 2013. The studies were located worldwide: 50% in Europe, 25% in North American, and 25% in other locations. Eleven studies had control groups. Twenty-two studies provided a definition of breastfeeding success; it ranged from 1 week to 24 weeks of breastfeeding. In 46 studies, we were able to determine the profession of the first author. In 42 of these studies, the first author was a plastic surgeon or had a plastic surgery affiliation [16,17,19,21–27,29–32,34,36–39,41,43,45–59,61–66].

Nine studies provided BMI; in 6 studies, the average BMI was ≥ 25, in the remainder of the studies the average BMI was <25. Two studies provided the disclosure that women received from surgeons on the impact of the surgery on future breastfeeding [40,60]. The disclosure was variable. It is unclear if the variability is due to different breastfeeding techniques, as the breast reduction techniques were not disclosed. Five studies (N = 86) provided reasons why women did not attempt to breastfeed, with the lack of support and encouragement being the predominant reason (72%) (S3 Table). Seven studies (N = 120) provided reasons for unsuccessful breastfeeding, with 55% of women reporting insufficient milk and 16% of women reporting reluctance and the lack of support (S3 Table).

In the 51 studies, there were 102 reported breast reduction techniques (see S4 Table for characteristics of each breast reduction technique). When the data were grouped based on the preservation of the column of subareolar parenchyma, 10 techniques had full preservation, 35 had a portion, 19 did not preserve any, and 38 did not have the preservation information. Ten studies provided the width of the pedicle; in all cases, it was ≥5 centimeters. In total, among the 102 reported techniques, there were 31 distinct breast reduction techniques.

Twenty-seven studies provided the satisfaction rate with breast reduction surgery; the median satisfaction rate among these studies was 92%. In 31 studies, the authors offered a conclusion of whether or not breast reduction surgery impacted breastfeeding; in 27 of these studies, the authors concluded breast reduction surgery did not impact breastfeeding [16–18,21,23,28–32,34,36–38,40,41,43,45–49,51,55,57,59,66].

### Quality assessment

**Fig 2:**
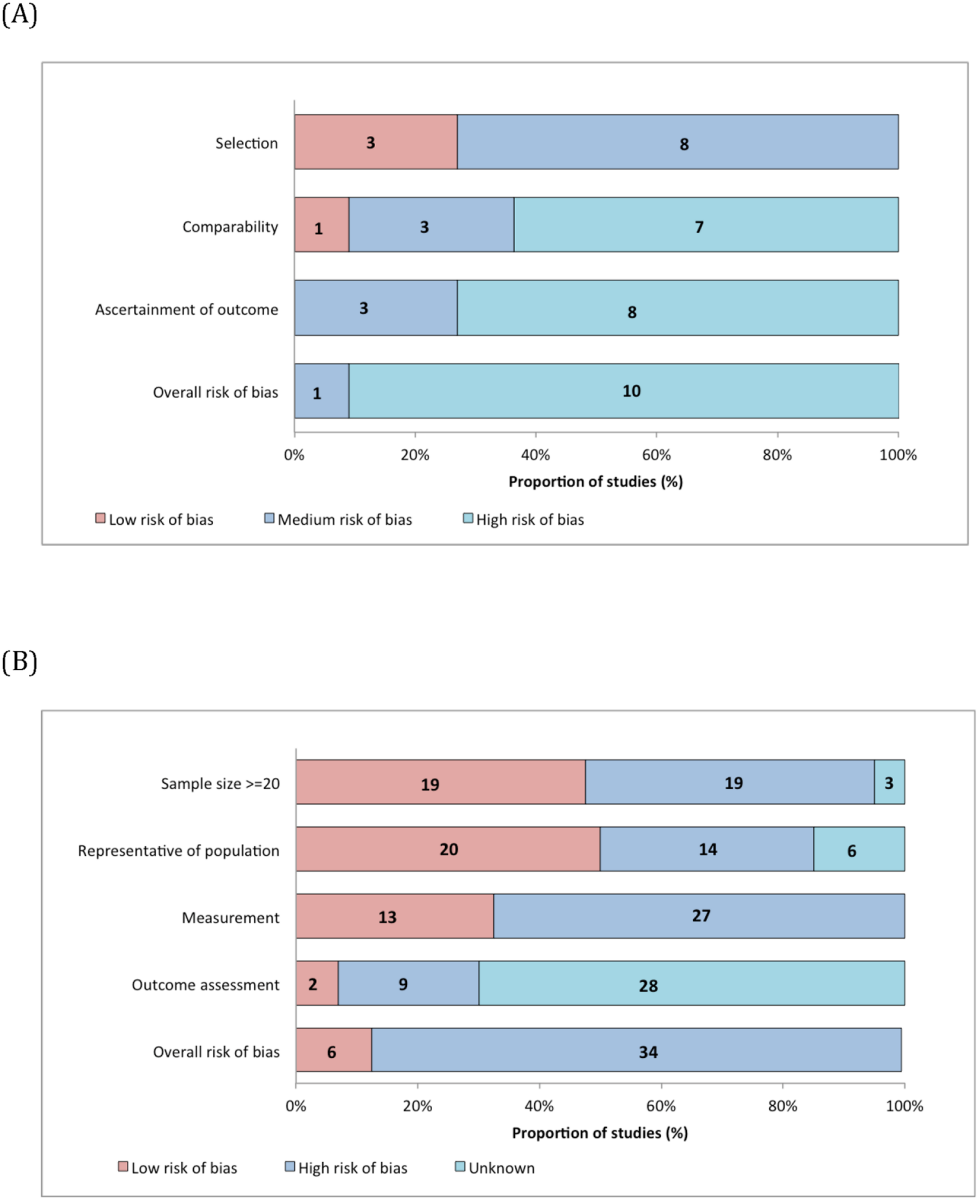
Risk of bias assessment. (A) Studies with control groups (B) studies without control groups.

Fig 2 shows the probability of bias for studies with and without control groups (see S5 Table for risk of bias calculation for each study). For studies without control groups, six had a low probability of bias and 34 had a high probability of bias. For studies with control groups, one had a medium probability of bias, and ten had a high probability of bias.

### Analysis

**Fig 3:**
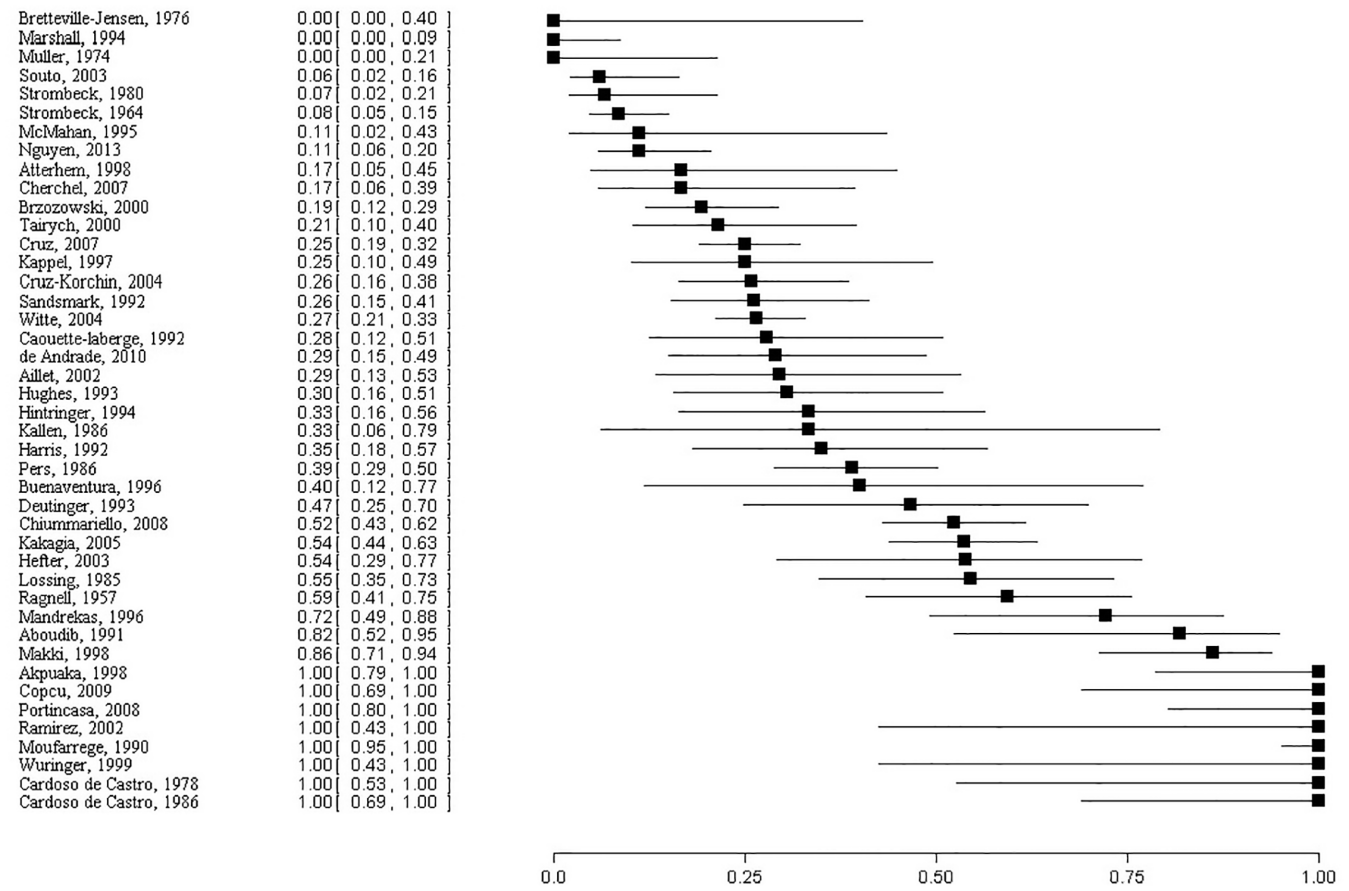
Forest plot of percent breastfeeding success with 95% confidence intervals.

Fig 3 shows the percent breastfeeding success and 95% confidence intervals for each of the 43 studies reporting women having children. The success ranged from no success in breastfeeding to complete success.

**Fig 4:**
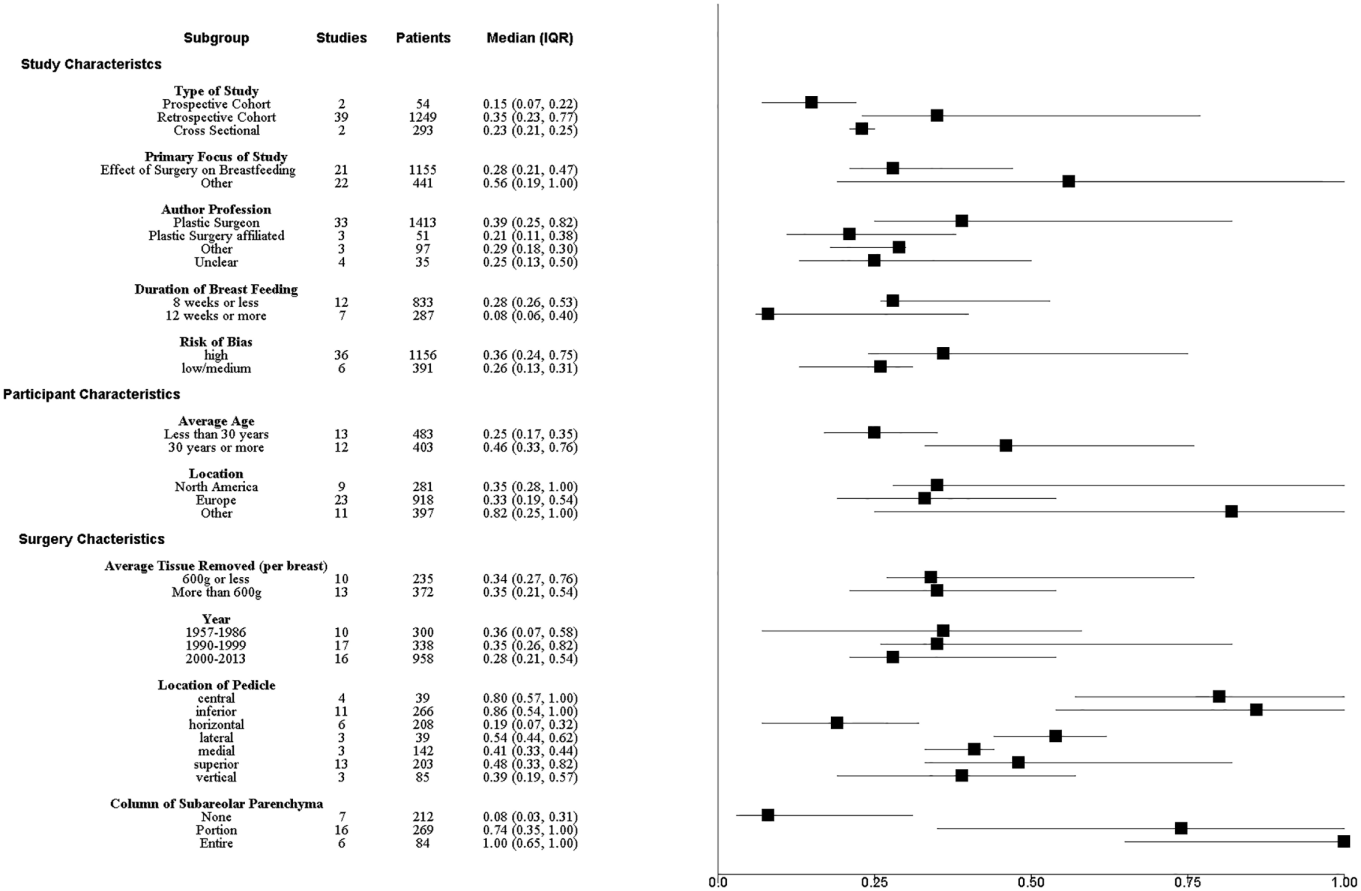
Forest plot of sensitivity analysis with median breastfeeding success and IQR.

Fig 4 shows the sensitivity analysis of each subgroup; the median percent success and IQRs are provided. Variables suggestive of higher study quality, including low risk of bias, prospective studies, breastfeeding ≥ 12 weeks, and focus of study on breastfeeding, had lower rates of successful breastfeeding. Studies with authors that were not plastic surgeons had a lower median breastfeeding success and a narrower IQR compared to studies authored by plastic surgeons. The amount of breast tissue removed and year did not appear to impact the rate of successful breastfeeding. Techniques with the inferior and central pedicle had greater breastfeeding success compared to other pedicle locations, but also had wide IQRs. Techniques that preserved the entire column of subareolar parenchyma had a greater median breast reduction success compared to techniques with no preservation and partial preservation.

The results of analyses with women who actually attempted breastfeeding as the denominator (instead of women with children) are provided in the supporting information (S1 Fig). Overall, the results were very similar, however, subgroup differences in the sensitivity analysis were more apparent.

## Discussion

**Fig 5:**
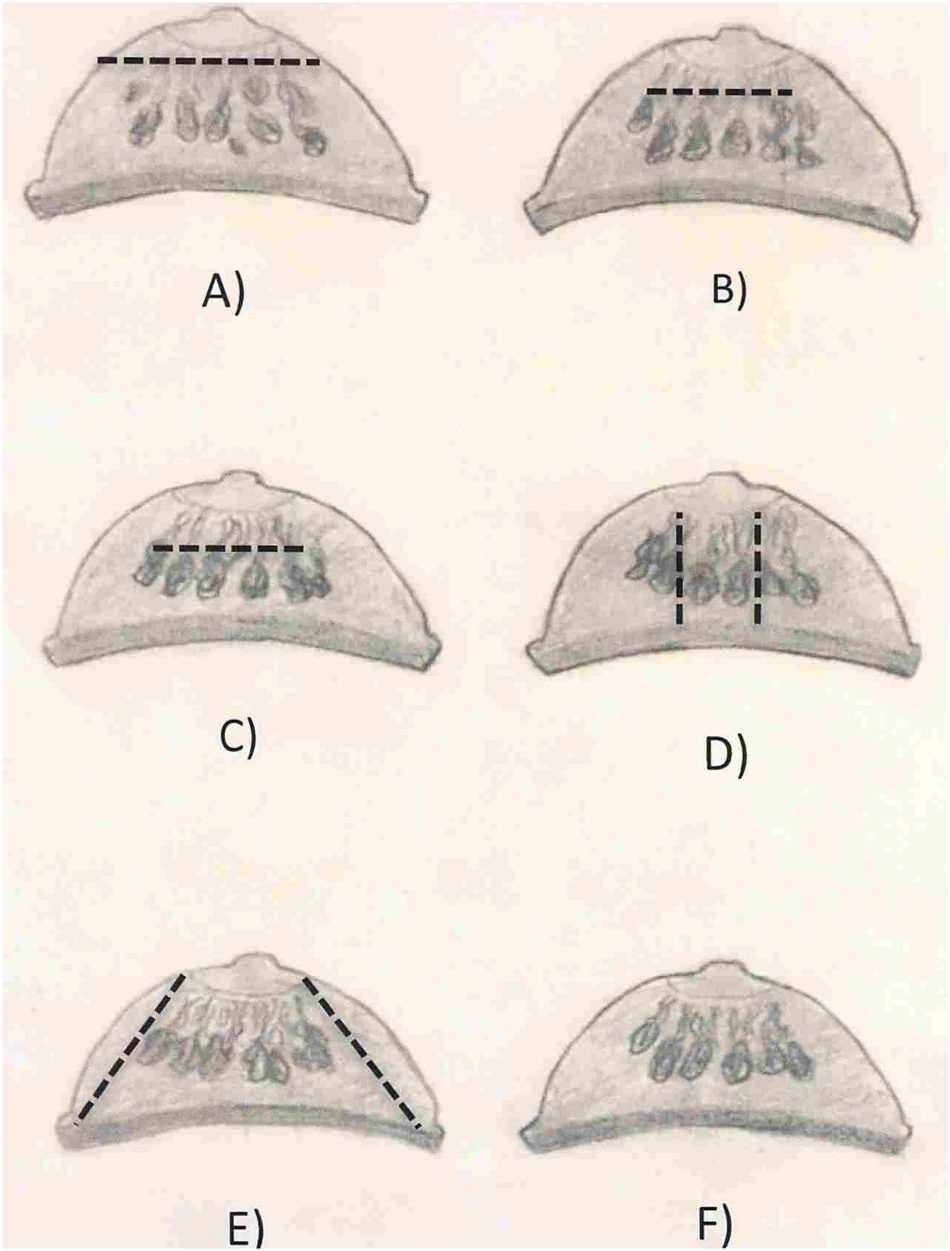
Cross section of breast with various breast reduction techniques. A) Free nipple transplant; B) Strombeck horizontal bipedicle; C) McKissock verticle bipedicle; D) Robbins inferior bipedicle; E) Moufarrege inferior pedicle; F) Ramirez superior bipedicle. ------ Transection. This figure illustrates the standard techniques, individual surgeon techniques may vary.

The impact of breast reduction surgery on breastfeeding can be thought of as a continuum ranging from complete transection of the column of subareolar parenchyma (free nipple transplant), resulting in no possibility of breastfeeding, to preservation of a portion of the column of subareolar parenchyma, resulting in variable breastfeeding success (pedicle techniques), to preservation of the entire column of subareolar parenchmya (pedicle techniques), resulting in potentially complete breastfeeding capability. Fig 5 illustrates this with 6 diverse techniques.

Preserving the entire column of the subareolar parenchyma can be achieved with a variety of breast reduction techniques. In the 1960s, Festge used a central mount approach with circular and tangential resection, which Hester refined in the 1980s to decrease the probability of nipple and skin flap necrosis [35,43,67]. In the late 1970s, Moufarrege developed the total posterior pedicle technique, based on the Robbins inferior pedicle technique. However, the resection is circumferential, instead of posterior to the pedicle, which allows the column of subareolar parenchyma to be preserved [51]. In the 1990s, Cardenas-Camarena designed a modified Skoog technique, which maintained the entire parenchyma beneath the superior lateral pedicle [24]. In 2002, Ramirez published a technique based on the vertical bipedicle approach; with an “owl shaped” inferior wedge resection that allows for the preservation of the entire column of subareolar parenchyma [57].

The extent to which these techniques are used is unclear; published studies provide statistics primarily based on pedicle location [68–70]. It is also unclear what disclosure women are given before surgery. Only two studies in this review provided this information; it was highly variable, from fully impacting breastfeeding to no impact. Considering the multitude of diverse techniques, unclear disclosure, and no guidelines on suitable breast reduction techniques for women of child bearing age, women contemplating breast reduction surgery appear to be at a considerable risk the surgery may impact their ability to breastfeed.

Psychosocial factors are commonly cited in published research as reasons for unsuccessful breastfeeding [11, 29,38,41,65]. However, the results from our systematic review point to breast reduction surgery itself affecting breastfeeding, as techniques that preserved the entire column of subareolar parenchyma had greater breastfeeding success. We also found the primary reason for inability to breastfeed was poor milk production, while the primary reason for not attempting breastfeeding was psychosocial factors.

To date, there has been only one systematic review on this topic. It concluded breast reduction surgery did not impact breastfeeding, and there was minimal difference among pedicle breast reduction techniques [11]. It was the first attempt to tackle this complex subject, however, without a comprehensive literature search, risk of bias assessment, credible statistical analysis, and a comprehensive sensitivity analysis, its conclusions are doubtful. The systematic review did, however, raise several important points, including advocating future researchers to use the WHO definition of successful breastfeeding, specifically 6 months of exclusive breastfeeding.

One limitation of the present review is the overall quality of studies; only 6 of the 51 studies had a low to medium risk of bias. It is doubtful this lack of quality impacts the overall conclusion; rather, the consistent trend seen among a diverse group of studies, with a high risk of bias, adds more credibility to the conclusion. Further, the high risk of bias among studies may lead to an overestimation of the breastfeeding success rate. Studies with a low to medium risk of bias had a lower median breastfeeding success rate. Compounding this, most studies used a definition of breastfeeding success of less than a month and milk demand increases over time. A further limitation of the present review is the often-incomplete descriptions of breast reduction techniques provided in studies, including pedicle width and the maintenance of the column of subareolar parenchyma. It would have been useful to further subdivide the studies based on the portion of the column of subareolar parenchyma maintained, but this was not possible in this review.

We recommend future well designed cohort studies to confirm our findings. Analysis of pedicle width would also be useful to determine if there is an optimal width for breastfeeding success. In addition, we suggest future studies to account for psychosocial factors and maternal BMI; without this, the impact of breast reduction surgery on breastfeeding will be unclear. It would also be valuable to delineate the most frequently used breast reduction techniques worldwide and their pedicle characteristics.

## Conclusion

In summary, techniques that keep the column of the subareolar breast parenchyma intact appear to provide a greater likelihood of breastfeeding success. Women considering breast reduction surgery should be told not only the name of the proposed breast reduction technique but its characteristics, including the extent the column of subareolar parenchyma will be preserved and pedicle width, to allow them to gain a better understanding of its impact on breastfeeding. High quality cohort studies would be invaluable to further ascertain the impact of pedicle characteristics on breastfeeding success.

## Supporting information

S1 Table PRISMA checklist. (DOC)

S2 Table Selected additional data of the studies. (DOCX)

S3 Table Reasons for not attempting to breastfeed and reasons for not breastfeeding successfully. (DOCX)

S4 Table Breast reduction technique characteristics. (DOCX)

S5 Table Risk of bias calculation. (DOCX)

S1 Text Search strategy. (DOCX)

S1 Fig Breastfeeding success and sensitivity analysis of women that attempted to breastfeed. (DOCX)
