# Supplementary material for: The impact of breast reduction surgery on breastfeeding: Systematic review of observational studies
Source: PLoS One. 2017 Oct 19;12(10):e0186591. doi: 10.1371/journal.pone.0186591 (PMC5648284; doi:10.1371/journal.pone.0186591)
Supplement: S2 Table — (DOCX) [file pone.0186591.s002.docx]

**S2 Table: Selected additional data of the studies**

| **Study** | **Average age at surgery** | **Average tissue removed per breast (g)** | **Satisfaction rate** | **BMI <25** | **Number attempted to breastfeed** | | **Number successfully breastfeeding** | |
| --- | --- | --- | --- | --- | --- | --- | --- | --- |
|  |  |  |  |  | **With reduction** | **Without reduction** | **With reduction** | **Without reduction** |
| Aboudib, 1991 | 27 | 481 | 94% | Not given | 11 | Not given | 9 | Not given |
| Aillet, 2002 | 17 | 530 | Not given | Not given | Not given | Not given | 5 | Not given |
| Akpuaka, 1998 | Not given | Not given | 100% | Not given | 10 | Not given | 10 | Not given |
| Atterhem, 1998 | 37 | Not given | Not given | No | 12 | Not given | 2 | Not given |
| Bretteville-Jensen, 1976 | Not given | Not given | 92% | Not given | 4 | Not given | 0 | Not given |
| Brzozowski, 2000 | Not given | 647 | 96% | Not given | 37 | Not given | 15 | Not given |
| Buenaventura, 1996 | 36 | 836 | Not given | No | 3 | Not given | 2 | Not given |
| Caouette-laberge, 1992 | Not given | 433 | Not given | Not given | 10 | Not given | 5 | Not given |
| Cardenas-Camarena, 2001 | 36 | 520 | Not given | Not given | Not given | Not given | 23 | Not given |
| Cardoso de Castro, 1978 | Not given | Not given | 91% | Not given | 3 | Not given | 3 | Not given |
| Cardoso de Castro, 1986 | Not given | 500 | 96% | Not given | 6 | Not given | 6 | Not given |
| Chen, 1997 | 33 | 480 | Not given | Not given | 12 | Not given | 9 | Not given |
| Cherchel, 2007 | 25 | 557 | 83% | Yes | 10 | Not given | 3 | Not given |
| Chiummariello, 2008 | 30 | Not given | Not given | Not given | Not given | Not given | 55 | Not given |
| Copcu, 2009 | 24 | 564 | Not given | Not given | 6 | Not given | 6 | Not given |
| Cruz-Korchin, 2004 | 26 | Not given | 100% | Not given | 37 | 92 | 15 | 36 |
| Cruz, 2007 | 28 | 610 | 100% | No | 102 | 93 | 41 | 38 |
| de Andrade, 2010 | Not given | Not given | Not given | Not given | 25 | 25 | 7 | 20 |
| Deutinger, 1993 | 23 | 855 | Not given | Not given | Not given | Not given | 7 | 5094 |
| Festge, 1960 | Not given | Not given | Not given | Not given | Not given | Not given | 6 | Not given |
| Hang-Fu, 1991 | 38 | 653 | 95% | Not given | 37 | Not given | 8 | Not given |
| Harris, 1992 | 25 | 605 | Not given | Not given | Not given | Not given | 7 | Not given |
| Hefter, 2003 | Not given | 601 | Not given | Not given | Not given | Not given | 7 | Not given |
| Hintringer, 1994 | 32 | 840 | 91% | Not given | Not given | Not given | 6 | 52 |
| Hughes, 1993 | Not given | Not given | Not given | Not given | 23 | Not given | 7 | Not given |
| Kakagia, 2005 | Not given | Not given | Not given | Not given | 74 | Not given | 52 | Not given |
| Kallen, 1986 | 35 | Not given | 86% | Not given | 2 | Not given | 1 | Not given |
| Kappel, 1997 | 22 | 428 | 88% | Yes | 12 | Not given | 4 | Not given |
| Lee, 2003 | 16 | 788 | 82% | Yes | 4 | Not given | 1 | Not given |
| Letertre, 2009 | 31 | 1340 | Not given | No | 5 | Not given | 4 | Not given |
| Lossing, 1985 | 34 | Not given | 92% | Not given | Not given | Not given | 12 | Not given |
| Makki, 1998 | 30 | 1036 | 68% | No | Not given | Not given | 31 | Not given |
| Mandrekas, 1996 | 33 | 870 | Not given | Not given | 18 | Not given | 13 | Not given |
| Marshall, 1994 | Not given | Not given | Not given | Not given | 27 | 314 | 0 | 164 |
| McMahan, 1995 | 18 | 674 | 94% | Not given | 4 | Not given | 1 | Not given |
| Moufarrege, 1990 | 30 | Not given | Not given | Not given | 54 | Not given | 54 | Not given |
| Muller, 1974 | Not given | Not given | 100% | Not given | Not given | Not given | 0 | Not given |
| Nguyen, 2013 | 29 | 566 | 96% | No | Not given | Not given | 8 | Not given |
| Pers, 1986 | 30 | 500 | 82% | Not given | Not given | Not given | 30 | Not given |
| Portincasa, 2008 | 48 | Not given | 81% | Not given | 11 | Not given | 11 | Not given |
| Ragnell, 1957 | Not given | 320 | Not given | Not given | Not given | Not given | 16 | 60 |
| Ramirez, 2002 | Not given | Not given | Not given | Not given | 2 | Not given | 2 | Not given |
| Sandsmark, 1992 | 30 | 545 | 98% | Not given | Not given | Not given | 11 | Not given |
| Sinno, 2013 | 33 | Not given | 93% | Not given | Not given | Not given | 166 | 160 |
| Souto, 2003 | 21 | Not given | 71% | Not given | 49 | 96 | 3 | 35 |
| Strombeck, 1964 | 35 | 350 | 99% | Not given | 3 | Not given | 1 | Not given |
| Strombeck, 1964 | Not given | Not given | Not given | Not given | 117 | 411 | 10 | 217 |
| Strombeck, 1981 | Not given | Not given | 79% | Not given | Not given | Not given | 2 | Not given |
| Tairych, 2000 | 29 | 610 | 89% | Not given | 15 | Not given | 6 | Not given |
| Witte, 2004 | Not given | Not given | Not given | Not given | 90 | Not given | 57 | Not given |
| Wuringer, 1999 | Not given | 712 | Not given | Not given | 2 | Not given | 2 | Not given |
