# Supplementary material for: The impact of breast reduction surgery on breastfeeding: Systematic review of observational studies
Source: PLoS One. 2017 Oct 19;12(10):e0186591. doi: 10.1371/journal.pone.0186591 (PMC5648284; doi:10.1371/journal.pone.0186591)
Supplement: S3 Table — (DOCX) [file pone.0186591.s003.docx]

**S3 Table: Reasons for not attempting to breastfeed and reasons for not breastfeeding successfully**

**The reasons for not attempting to breastfeed (in the five studies that provided this data)**

| **Study** | **Total # of women** | **Insufficient milk** | **Lack of desire / support** | **Medical advice** | **Maternal medical issues** | **Returning to work / too busy** | **Infant issues** |
| --- | --- | --- | --- | --- | --- | --- | --- |
| Cherchel, 2007 | 8 |  | 6 | 1 |  |  | 1 |
| Brzozowski, 2000 | 41 |  | 28 | 9 | 1 | 2 | 1 |
| Caouette-laberge, 1992 | 9 |  | 6 | 1 |  |  | 2 |
| Kakagia, 2005 | 23 |  | 15 |  | 8 |  |  |
| Kappel, 1997 | 5 |  | 1 |  | 3 |  | 1 |
| Total | 86 | 0 | 56 | 11 | 12 | 2 | 5 |
| Percentage | 100% | 0% | 65% | 13% | 14% | 2% | 6% |

**The reasons for not breastfeeding successfully (in the seven studies that provided this data)**

| **Study** | **Total # of women** | **Insufficient milk** | **Lack of desire / support** | **Medical advice** | **Maternal medical issues** | **Returning to work / too busy** | **Infant issues** |
| --- | --- | --- | --- | --- | --- | --- | --- |
| Cherchel, 2007 | 8 | 3 |  |  | 1 | 3 | 1 |
| Souto, 2003 | 45 | 35 | 3 | 2 | 1 | 3 | 1 |
| Brzozowski, 2000 | 14 | 5 | 1 | 1 | 2 |  | 5 |
| Caouette-laberge, 1992 | 7 | 2 |  |  | 1 | 2 | 2 |
| Kakagia, 2005 | 22 |  | 15 |  | 7 |  |  |
| Kappel, 1997 | 3 |  |  |  | 1 |  | 2 |
| Sandsmark, 1992 | 21 | 21 |  |  |  |  |  |
| Total | 120 | 66 | 19 | 3 | 13 | 8 | 11 |
| Percentage | 100% | 55% | 16% | 3% | 11% | 7% | 9% |
