# Supplementary material for: The impact of breast reduction surgery on breastfeeding: Systematic review of observational studies
Source: PLoS One. 2017 Oct 19;12(10):e0186591. doi: 10.1371/journal.pone.0186591 (PMC5648284; doi:10.1371/journal.pone.0186591)
Supplement: S4 Table — (DOCX) [file pone.0186591.s004.docx]

**S4 Table: Breast reduction technique characteristics**

|  | **Technique used** | **Pedicle location** | **Column of subareolar parenchyma preserved** | **Pedicle width (cm)** |
| --- | --- | --- | --- | --- |
| Aboudib, 1991 | Pitanguy | Superior | Portion | Not given |
| Aillet, 2002 | Not given | Not given | Not given | Not given |
|  | Not given | Not given | Not given | Not given |
|  | Not given | Inferior | Not given | Not given |
|  | Not given | Lateral | Not given | Not given |
|  | Not given | Superior | Not given | Not given |
| Akpuaka, 1998 | Robbins | Inferior | Portion | Not given |
| Atterhem, 1998 | Nipple areola graft | free nipple | None | Not given |
|  | Regnault | Superior | Portion | Not given |
|  | Skoog | Lateral | None | Not given |
|  | Strombeck | Horizontal | None | Not given |
| Bretteville-Jensen, 1976 | Bretteville-Jensen | Vertical | None | Not given |
| Brzozowski, 2000 | Hurst (modified Robertson) | Inferior | None | 8 cm |
|  | Robbins | Inferior | Portion | Not given |
| Buenaventura, 1996 | Not given | Central | Not given | Not given |
|  | Not given | Inferior | Not given | Not given |
|  | Nipple areola graft | free nipple | Not given | Not given |
| Caouette-laberge, 1992 | Regnault | Superior | Portion | Not given |
|  | Robbins | Inferior | Portion | Not given |
| Cardenas-Camarena, 2001 | Cardenas-Camarena | superior/lateral | Entire | 6 - 8 cm |
| Cardoso de Castro, 1978 | Pitanguy | Superior | Portion | Not given |
| Cardoso de Castro, 1986 | Pitanguy | Superior | Portion | Not given |
| Chen, 1997 | Lejour | Superior | Portion | Not given |
| Cherchel, 2007 | Lejour | Superior | Portion | Not given |
| Chiummariello, 2008 | Not given | Inferior | Not given | Not given |
|  | Not given | lateral | Not given | Not given |
|  | Not given | medial | Not given | Not given |
|  | Not given | superior | Not given | Not given |
| Copcu, 2009 | Copcu | inferior | Entire | 6 cm |
| Cruz-Korchin, 2004 | Hall-Findlay | medial | Portion | 6 – 8 cm |
| Cruz, 2007 | Not given | inferior | Not given | Not given |
|  | Not given | medial | Not given | Not given |
|  | Not given | superior | Not given | Not given |
| de Andrade, 2010 | Not given | Not given | Not given | Not given |
| Deutinger, 1993 | McKissock | vertical | Portion | Not given |
|  | Pitanguy | superior | Portion | Not given |
|  | Strombeck | horizontal | None | Not given |
| Festge, 1960 | Festge | central | Entire | Not given |
| Hang-Fu, 1991 | McKissock | vertical | Portion | Not given |
|  | Regnault modified | superior | Entire | Not given |
|  | Nipple areola graft | free nipple | None | Not given |
|  | Regnault | superior | Portion | Not given |
|  | Robbins | inferior | Portion | Not given |
|  | Strombeck | horizontal | None | Not given |
| Harris, 1992 | Robbins | inferior | Portion | Not given |
| Hefter, 2003 | Hefter | lateral | Entire | Not given |
| Hintringer, 1994 | Not given | superior | Not given | Not given |
| Hughes, 1993 | Not given | Not given | Not given | Not given |
| Kakagia, 2005 | Not given | inferior | Not given | Not given |
|  | Not given | superior | Not given | Not given |
|  | Strombeck | horizontal | None | Not given |
| Kallen, 1986 | Strombeck | horizontal | None | Not given |
| Kappel, 1997 | Biessenberger modified | central | Entire | Not given |
|  | Weiner | superior | Portion | Not given |
| Lee, 2003 | Not given | inferior | Not given | Not given |
|  | Not given | superior | Not given | Not given |
|  | Not given | vertical | Not given | Not given |
|  | Nipple areola graft | free nipple | Not given | Not given |
| Letertre, 2009 | Robbins | inferior | Portion | Not given |
| Lossing, 1985 | Strombeck modified | superior | Portion | Not given |
| Makki, 1998 | Not given | inferior | Not given | Not given |
| Mandrekas, 1996 | Robbins | inferior | Portion | 4 – 5 cm |
| Marshall, 1994 | McKissock | vertical | Portion | Not given |
|  | Nipple areola graft | free nipple | None | Not given |
|  | Robbins | inferior | Portion | Not given |
|  | Strombeck | horizontal | Not given | Not given |
| McMahan, 1995 | Not given | horizontal | Not given | Not given |
|  | Not given | inferior | Not given | Not given |
|  | Not given | lateral | Not given | Not given |
|  | Not given | superior | Not given | Not given |
|  | Not given | vertical | Not given | Not given |
|  | Nipple areola graft | free nipple | Not given | Not given |
| Moufarrege, 1990 | Moufarrege | inferior | Entire | 6 cm |
| Muller, 1974 | Strombeck | horizontal | None | Not given |
| Nguyen, 2013 | Not given | Not given | Not given | Not given |
| Pers, 1986 | McKissock | vertical | Portion | Not given |
|  | Pers & Bretteville-Jensen | vertical | Portion | Not given |
| Portincasa, 2008 | Hester | inferior | Entire | Not given |
|  | Robbins | inferior | Portion | Not given |
|  | Weiner | superior | Portion | Not given |
| Ragnell, 1957 | Not given | central | Not given | Not given |
|  | Not given | Not given | Not given | Not given |
| Ramirez, 2002 | Ramirez | central | Entire | 4 – 5 cm |
| Sandsmark, 1992 | Blomqvist | superior/lateral | Portion | Not given |
|  | Holmstrum | superior/medial | Portion | 6 - 8 cm |
|  | Nipple areola graft | free nipple | None | Not given |
|  | Orlando | superior | None | Not given |
|  | Robbins | inferior | Portion | Not given |
|  | Strombeck | horizontal | None | Not given |
| Sinno, 2013 | Moufarrege | inferior | Entire | 6 cm |
| Souto, 2003 | Not given | Not given | Not given | Not given |
| Strombeck, 1964 | Strombeck | horizontal | None | Not given |
| Strombeck, 1964 | Strombeck | horizontal | None | Not given |
| Strombeck, 1980 | Strombeck | horizontal | None | Not given |
| Tairych, 2000 | Georgiade | inferior | Portion | 7 – 9 cm |
|  | Lexer-Kraske | central | Not given | G |
|  | McKissock | vertical | Portion | Not given |
|  | Nipple areola graft | free nipple | None | Not given |
|  | Pitanguy and Peixoto | superior | Portion | Not given |
| Witte, 2004 | Lassus modified | superior | Portion | Not given |
|  | Strombeck | horizontal | Not given | Not given |
| Wuringer, 1999 | Wuringer | central | Portion | Not given |

**References:**

Biessenberger modified: Kappel RM, Dijkstra R, Storm van Leeuwen JB, Houpt P, Kuyper M. Nipple sensitivity and lactation in two methods of breast reduction. Eur J Plast Surg 1997;20:60-5.

Blomqvist: Blomqvist G, Alberius P. Nipple-Areola Transposition by the Superolateral-rotation pedicle technique in reduction mammoplasty: surgical description. Ann of Plast Surg 1990;24:475-80.

Bretteville-Jensen: Bretteville-Jensen G. Reduction mammoplasty with a vertical bipedicle and transverse scar: a follow up. Br J Plast Surg 1976;29:142-49.

Cardenas-Camarena: Cardenas-Camarena L, Vergara R. Reduction mammoplasty with superior dermoglandular pedicle: another alternative. Plast Reconstr Surg 2001;107:693-99.

Copcu: Copcu E. A versatile breast reduction technique: conical plicated central U shaped (COPCUs) mammaplasty. Ann Surg Innov Res 2009;3:1-11.

Festge: Festge O. Eine abgewandelte technik bei der mammaplastik zur vermeidung von mamillen und gewebsnekrosen. Zentralbl Chir 1960; 50:2365-70.

Georgiade: Georgiade GS, Riefkohl RD, Georgiade NG. The inferior dermal-pyramidal type breast reduction: long term evaluation. Ann of Plast Surg 1989;23:203-11.

Hall-Findlay: Hall-Findlay EJ. A simplified vertical reduction mammaplasty: shortening the learning curve. Plast Reconstr Surg 1999;104:748-59.

Hefter: Hefter W, Lindholm P, Elvenes OP. Lactation and breast-feeding ability following lateral pedicle mammoplasty. Br J Plast Surg 2003;56:746-51.

Hester: Hester TR, Bostwick J, Miller L, Cunningham SJ. Breast reduction utilizing the maximally vascularized central breast pedicle. Plast Reconstr Surg 1985;76:890-900.

Holmstrom: Holmstrom H, Lossing C. Reduction mammaplasty with a sliding nipple technique. Scand J Plast Recontsr Hand Surg 1990;24:245-252.

Hurst: Hurst LN, Evans HB, and Murray KA. Inferior flap reduction mammoplasty with pedicled nipple. Ann Plast Surg 1983;10:483-87.

Lassus: Lassus C. A 30-year experience with vertical mammaplasty. Plast Reconstr Surg 1996;97:373-80.

Lejour M, Abboud M. Vertical mammoplasty without inframammary scar and with breast liposuction. *Perspectives plast surg* 1990;4:67-90.

Lexer-Kraske: Santoni-Gariu P. A history of plastic surgery. Berlin: Springer 2007. 335-6.

McKissock: McKissock, PK. Reduction mammoplasty with a vertical dermal flap. Plast Reconstr Surg 1972;49:245-52.

Moufarrege: Moufarrege R. The total dermoglandular pedicle mammoplasty. In: Georgiade NG, editor. Aesthetic surgery of the breast. Philadelphia, US: Saunders, 1990. 371-86.

Nipple areola transposition: not required

Orlando: Orlando JC, Gunthrie RH. The superomedial dermal pedicle for nipple transposition. Br J Plast Surg 1975;28:42-5.

Pers and Bretteville-Jenseon: Pers M, Nielsen IM, Gerner N. Results following reduction mammoplasty as evaluated by the patients. Annals Plast Surg 1986;16:449-455.

Peixeto: Peixoto G: Reduction mammaplasty: A personal technique. Plast Reconstr Surg 1980;65:217-26.

Pitanguy: Pitanguy I. Surgical treatment of breast hypertrophy. Br J Plast Surg. 1967;20:78-85.

Ramirez: Ramirez O. Reduction mammoplasty with the “owl” incision and no undermining. Plast Reconstr Surg 2002;109:512-22.

Regnault: Regnault P. Reduction mammaplasty by the “B” technique. Plast Reconstr Surg 1974;53:19–24.

Regnault modified: Hang-Fu L. Subjective comparison of six different reduction mammoplasty procedures. Aesthetic Plast Surg 1991;15:297-302.

Robbins: Restifo F. The robbins inferior pedicle reduction mammaplasty. In Shiffman M, editor. Mastoplexy and breast reduction: principles and practice. Berlin: Springer 2009.

Skoog: Skoog T. A technique of breast reduction: transposition of the nipple on a cutaneous vascular pedicle. Acta Chir Scand 1963:126:453-65.

Strombeck: Strombeck JO. Reduction Mammaplasty. In Gibson T, editor. Modern Trends in Plastic Surgery 1. London, UK: Butterworths, 1964. 237-55.

Stombeck modified (Lossing): Lossing C, Holmstrom H. [Mammary hyperplasia with preserved ability to suckle]. Lakartidningen 1985;82:2878-81.

Weiner: Weiner DL, Dolich BH, Miclat MI. Reduction mammoplasty using superior pedicle technique. Aesthet Plast Surg 1982;6:7-14.

Wuringer: Wuringer E. Refinement of the central pedicle breast reduction by application of the ligamentous suspension. Plast Reconstr Surg 1999;103:1400-10.
