# Supplementary material for: The impact of breast reduction surgery on breastfeeding: Systematic review of observational studies
Source: PLoS One. 2017 Oct 19;12(10):e0186591. doi: 10.1371/journal.pone.0186591 (PMC5648284; doi:10.1371/journal.pone.0186591)
Supplement: S5 Table — (DOCX) [file pone.0186591.s005.docx]

**S5 Table: Risk of bias calculation**

Studies without control groups:

| **Study** | **Adequate sample size** | **Representative of population** | | **Measurement bias** | **Outcome assessment bias** | **Risk of Bias (total score)** |
| --- | --- | --- | --- | --- | --- | --- |
|  | **# of women with children** | **Subset excluded** | **Participation rate ≥50%** | **Breastfeeding success defined** | **Follow up ≥24 weeks** |  |
| Aboudib, 1991 | 11 | Yes | 20% | No | Not given | High (0) |
| Aillet, 2002 | 17 | Yes | 60% | No | Not given | HIgh (0) |
| Akpuaka, 1998 | 10 | No | 100% | No | Not given | HIgh (1) |
| Atterhem, 1998 | 12 | No | 88% | No | Not given | High (1) |
| Bretteville-Jensen, 1976 | 4 | No | 71% | No | Not given | High (1) |
| Brzozowski, 2000 | 78 | No | 100% | Yes | 2 weeks | Low (3) |
| Buenaventura, 1996 | 5 | No | 52% | No | Not given | High (1) |
| Caouette-laberge, 1992 | 18 | No | 40% | Yes | 3 weeks | High (1) |
| Cardenas-Camarena, 2001 | Not given | No | 100% | No | Not given | HIgh (1) |
| Cardoso de Castro, 1978 | 3 | Not given | not given | No | Not given | HIgh (0) |
| Cardoso de Castro, 1986 | 6 | Yes | 100% | No | Not given | HIgh (0) |
| Chen, 1997 | 12 * | No | 100% | No | Not given | High (1) |
| Chiummariello, 2008 | 105 | Yes | 80% | Yes | 3 weeks | HIgh (2) |
| Copcu, 2009 | 6 | No | 100% | Yes | 24 weeks | Low (3) |
| Festge, 1960 | Not given | No | 100% | No | Not given | High (1) |
| Hang-Fu, 1991 | 37 * | No | 40% | No | Not given | HIgh (1) |
| Harris, 1992 | 20 | No | 93% | Yes | 8 weeks | Low (3) |
| Hefter, 2003 | 13 | Yes | not given | Yes | 8 weeks | High (0) |
| Hughes, 1993 | 23 | Not given | not given | Yes | Not given | HIgh (1) |
| Kakagia, 2005 | 97 | No | not given | Yes | 3 weeks | High (2) |
| Kallen, 1986 | 3 | No | 85% | No | Not given | High (1) |
| Kappel, 1997 | 16 | No | not given | No | Not given | High (0) |
| Lee, 2003 | 4 * | Yes | 23% | No | Not given | High (0) |
| Letertre, 2009 | 5 * | Yes | 100% | No | Not given | High (0) |
| Lossing, 1985 | 22 | Not given | 25% | Yes | 4 weeks | High (2) |
| Makki, 1998 | 36 | No | 55% | No | Not given | High (2) |
| Mandrekas, 1996 | 18 | No | 100% | No | Not given | High (1) |
| McMahan, 1995 | 9 | Yes | 56% | No | Not given | High (0) |
| Moufarrege, 1990 | 20 | No | 100% | No | Not given | High (2) |
| Muller, 1974 | 10 | Not given | 100% | No | Not given | High (0) |
| Nguyen, 2013 | 72 | Yes | 49% | No | Not given | High (0) |
| Pers, 1986 | 77 | No | 91% | No | Not given | High (2) |
| Portincasa, 2008 | 11 | No | 100% | No | Not given | High (1) |
| Ramirez, 2002 | 2 | No | 100% | No | Not given | High (1) |
| Sandsmark, 1992 | 42 | No | 94% | Yes | Not given | Low (3) |
| Strombeck, 1964 | 118 | Not given | not given | Yes | 4 weeks | High (2) |
| Strombeck, 1980 | 30 | Yes | not given | Yes | 24 weeks | Low (3) |
| Tairych, 2000 | 28 | No | 48% | No | 24 weeks | High (2) |
| Witte, 2004 | 215 | No | 59% | Yes | 1 week | Low (3) |
| Wuringer, 1999 | 2 | No | 100% | No | Not given | High (1) |

Studies with control groups:

| Study | Selection Bias^1^ | | | | Comparability bias^2^ | | Outcome bias^3^ | | | Risk of Bias |
| --- | --- | --- | --- | --- | --- | --- | --- | --- | --- | --- |
|  | 1 | 2 | 3 | 4 | 1 | 2 | 1 | 2 | 3 |  |
| Cherchel, 2007^35^ | Unclear | Yes | Records | Yes | No | No | Self report | Yes | Not given | High |
| Cruz-Korchin, 2004^38^ | Unclear | Yes | Records | Yes | No | Yes | Self report | No | Not given | High |
| Cruz, 2007^11^ | Unclear | Yes | Records | Yes | No | Yes | Self report | No | Not given | High |
| de Andrade, 2010^39^ | Unclear | Yes | Examination | Yes | No | Yes | Assessed | No | No | High |
| Deutinger, 1993^40^ | Somewhat | No | Records | Yes | No | No | Self report | Not given | Not given | High |
| Hintringer, 1994^45^ | Somewhat | Yes | Records | Yes | No | No | Self report | Not given | Yes | High |
| Marshall, 1994^14^ | Unclear | Yes | Unclear | Yes | No | No | Assessed | No | Yes | High |
| Ragnell, 1957^62^ | Somewhat | Yes | Records | Yes | No | No | Not given | Yes | Not given | High |
| Sinno, 2013^65^ | Somewhat | Yes | Records | Yes | No | No | Self report | Yes | Yes | High |
| Souto, 2003^12^ | Unclear | Yes | Records | Yes | Yes | Yes | Self report | Yes | Yes | Medium |
| Strombeck, 1964^66^ | Unclear | Yes | Records | Yes | No | No | Self report | No | Not given | High |

^1^Selection bias column headings:

1. Representativeness of the exposed cohort
2. Non exposed cohort drawn from the same community as the exposed cohort
3. Ascertainment of exposure
4. Demonstration that outcome of interest was not present at start

^2^ Comparability bias column headings:

1. Exposed and non exposed cohort were compared on the most important factor (psychosocial support)
2. Exposed and non exposed cohort were compared on any secondary factor

^3^ Outcome bias column headings:

1. Assessment of outcome
2. Was follow up long enough for outcomes to occur (6 months)
3. Adequacy of follow up of cohort (prospective cohort: loss to follow up ≤ 10%, retrospective cohort: followed ≥ 12 months
