# Supplementary material for: The impact of breast reduction surgery on breastfeeding: Systematic review of observational studies
Source: PLoS One. 2017 Oct 19;12(10):e0186591. doi: 10.1371/journal.pone.0186591 (PMC5648284; doi:10.1371/journal.pone.0186591)

**S1 Text: Search strategy**

**Medline (Pubmed) Searched December 23, 2014**

Database: Ovid MEDLINE(R) In-Process & Other Non-Indexed Citations and Ovid MEDLINE(R) <1946 to Present>
Search Strategy:
--------------------------------------------------------------------------------
1     exp Mammaplasty/ or breast [reduction.mp](http://reduction.mp/). (11798)
2     [segmentectomy.mp](http://segmentectomy.mp/). or exp Mastectomy, Segmental/ (8349)
3     ((mastectom* adj2 local excision) or (limited resection adj2 mastectom*)).mp. (52)
4     partial mastectom*.mp. (464)
5     mammoplast*.mp. (1146)
6     breast plastic [operation.mp](http://operation.mp/). (0)
7     exp Breast/ or breast*.mp. (384193)
8     exp Surgery, Plastic/ or plastic surger*.mp. (34809)
9     6 or 8 (34809)
10     7 and 9 (3936)
11     1 or 2 or 3 or 4 or 5 or 10 (23604)
12     exp Breast Feeding/ or breast feed*.mp. (32240)
13     Lactation/ (33618)
14     breastfeed*.mp. (15528)
15     suckl*.mp. [mp=title, abstract, original title, name of substance word, subject heading word, keyword heading
word, protocol supplementary concept word, rare disease supplementary concept word, unique identifier] (13822)
16     (breastfed or breast fed).mp. [mp=title, abstract, original title, name of substance word, subject heading word,
keyword heading word, protocol supplementary concept word, rare disease supplementary concept word, unique identifier]
(9105)
17     12 or 13 or 14 or 15 or 16 (80502)
18     11 and 17 (196)
19     case report*.ti. (181322)
20     18 not 19 (193)

**EMBASE Searched December 23, 2014**

Database: Embase <1974 to 2014 December 22>
Search Strategy:
--------------------------------------------------------------------------------
1     exp "breast reconstruction"/ or breast [reduction.mp](http://reduction.mp/). (14940)
2     mammaplast*.mp. (2318)
3     breast plastic [operation.mp](http://operation.mp/). (0)
4     exp partial mastectomy/ (9029)
5     exp Breast/ or breast*.mp. (539251)
6     exp exp plastic surgery/ or plastic surger*.mp. (62522)
7     5 and 6 (4704)
8     1 or 2 or 3 or 4 or 7 (25734)
9     exp "breast feeding"/ or breast feed*.mp. (40512)
10     "lactation"/ (39708)
11     breastfeed*.mp. (17878)
12     suckl*.mp. [mp=title, abstract, subject headings, heading word, drug trade name, original title, device
manufacturer, drug manufacturer, device trade name, keyword] (13012)
13     (breastfed or breast fed).mp. [mp=title, abstract, subject headings, heading word, drug trade name, original
title, device manufacturer, drug manufacturer, device trade name, keyword] (10349)
14     9 or 10 or 11 or 12 or 13 (92732)
15     8 and 14 (262)
16     case report*.ti. (223517)
17     15 not 16 (255)

**EBM ALL Searched December 23, 2014**

Database: EBM Reviews - Cochrane Database of Systematic Reviews <2005 to November 2014>, EBM Reviews - ACP Journal Club<1991 to December 2014>, EBM Reviews - Database of Abstracts of Reviews of Effects <4th Quarter 2014>, EBM Reviews -Cochrane Central Register of Controlled Trials <November 2014>, EBM Reviews - Cochrane Methodology Register <3rd Quarter2012>, EBM Reviews - Health Technology Assessment <4th Quarter 2014>, EBM Reviews - NHS Economic Evaluation Database<4th Quarter 2014>
Search Strategy:
--------------------------------------------------------------------------------
1     exp Mammaplasty/ or breast [reduction.mp](http://reduction.mp/). (244)
2     [segmentectomy.mp](http://segmentectomy.mp/). or exp Mastectomy, Segmental/ (424)
3     ((mastectom* adj2 local excision) or (limited resection adj2 mastectom*)).mp. (18)
4     partial mastectom*.mp. (161)
5     mammoplast*.mp. (42)
6     breast plastic [operation.mp](http://operation.mp/). (0)
7     exp Breast/ or breast*.mp. (24134)
8     exp Surgery, Plastic/ or plastic surger*.mp. (426)
9     6 or 8 (426)
10     7 and 9 (46)
11     1 or 2 or 3 or 4 or 5 or 10 (869)
12     exp Breast Feeding/ or breast feed*.mp. (2170)
13     Lactation/ (426)
14     breastfeed*.mp. (1567)
15     suckl*.mp. [mp=ti, ab, tx, kw, ct, ot, sh, hw] (146)
16     (breastfed or breast fed).mp. [mp=ti, ab, tx, kw, ct, ot, sh, hw] (1023)
17     12 or 13 or 14 or 15 or 16 (3560)
18     11 and 17 (2)
19     case report*.ti. (177)
20     18 not 19 (2)

**Proquest Dissertations and Theses Full Text. Searched December 23, 2014**

all(((breast* or mammar*) and ("plastic surger*" or "Plastic operation*" or "local excision*" or "limited resection*"))) OR all(mammoplast* or "partial mastect*" or "breast reduct*" or segmentectomy ) AND (suckl* OR breastfeed* OR breastfed* OR "breast feed*" OR "breast fed*" OR lactates OR lactate OR lactation OR lactating) =37

**SCOPUS Searched December 23, 2014**

((TITLE-ABS-KEY((((breast* or mammar*) and ("plastic surger*" or "Plastic operation*" or "local excision*" or "limited resection*")))) OR TITLE-ABS-KEY(mammoplast* or "partial mastect*" or "breast reduct*" or segmentectomy) AND TITLE-ABS-KEY(suckl* OR breastfeed* OR breastfed* OR "breast feed*" OR "breast fed*" OR lactates OR lactate OR lactation OR lactating))) and not (TITLE(case report)) = 271

**CINAHL Searched December 23, 2014**


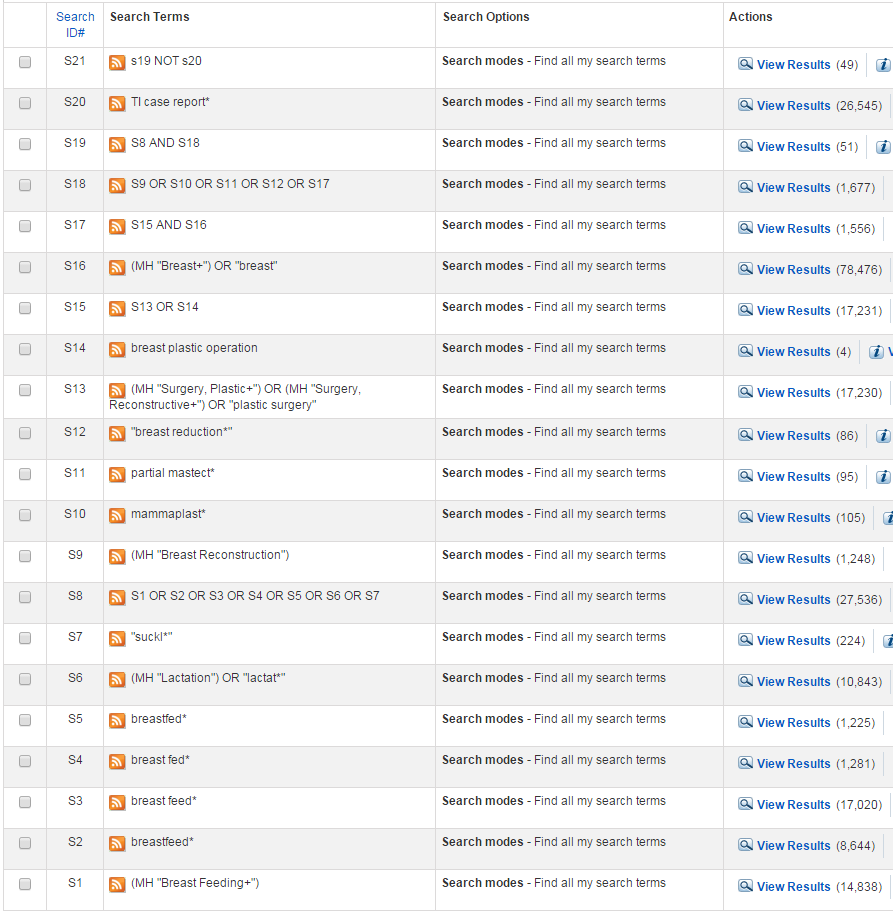

Supplement: S1 Text — (DOCX) [file pone.0186591.s006.docx]
