# Supplementary material for: The impact of breast reduction surgery on breastfeeding: Systematic review of observational studies
Source: PLoS One. 2017 Oct 19;12(10):e0186591. doi: 10.1371/journal.pone.0186591 (PMC5648284; doi:10.1371/journal.pone.0186591)
Supplement: S1 Fig — (DOCX) [file pone.0186591.s007.docx]

**S1 Figure: Breastfeeding success and sensitivity analysis of women that attempted to breastfeed**

Forest plot of percent breastfeeding success with 95% confidence intervals (denominator is women that attempted to breastfeed)

**
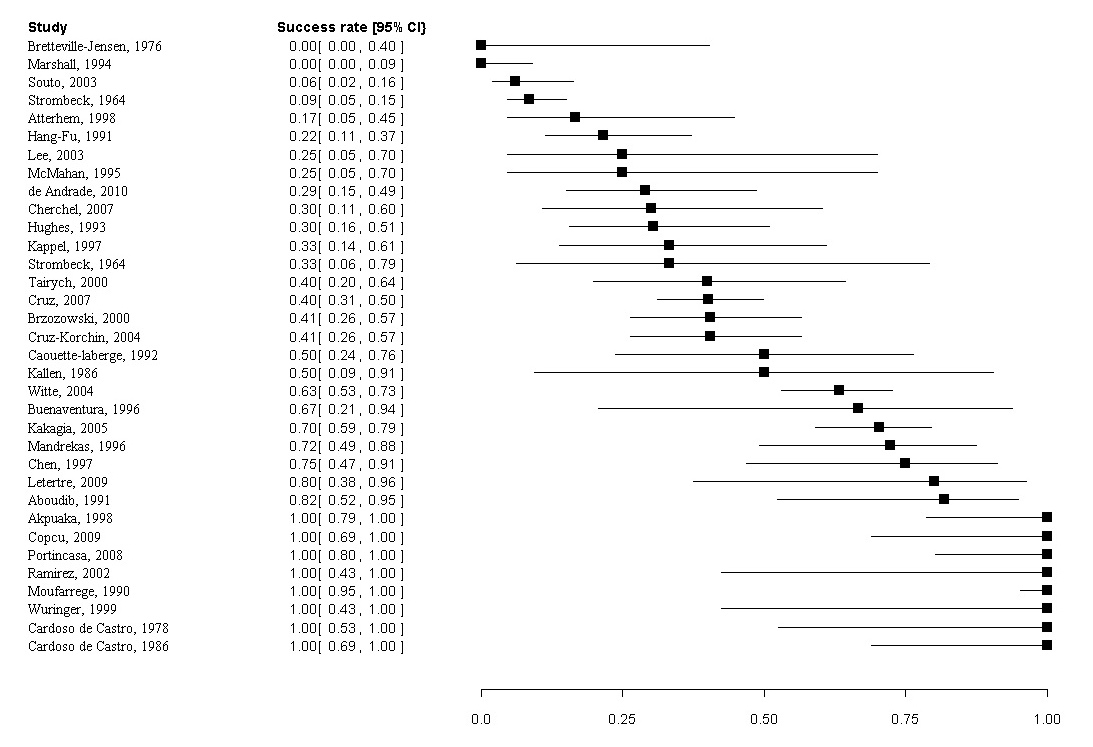
**

Forest plot of sensitivity analysis with median breastfeeding success and interquartile range (denominator is women that attempted to breastfeed)

**
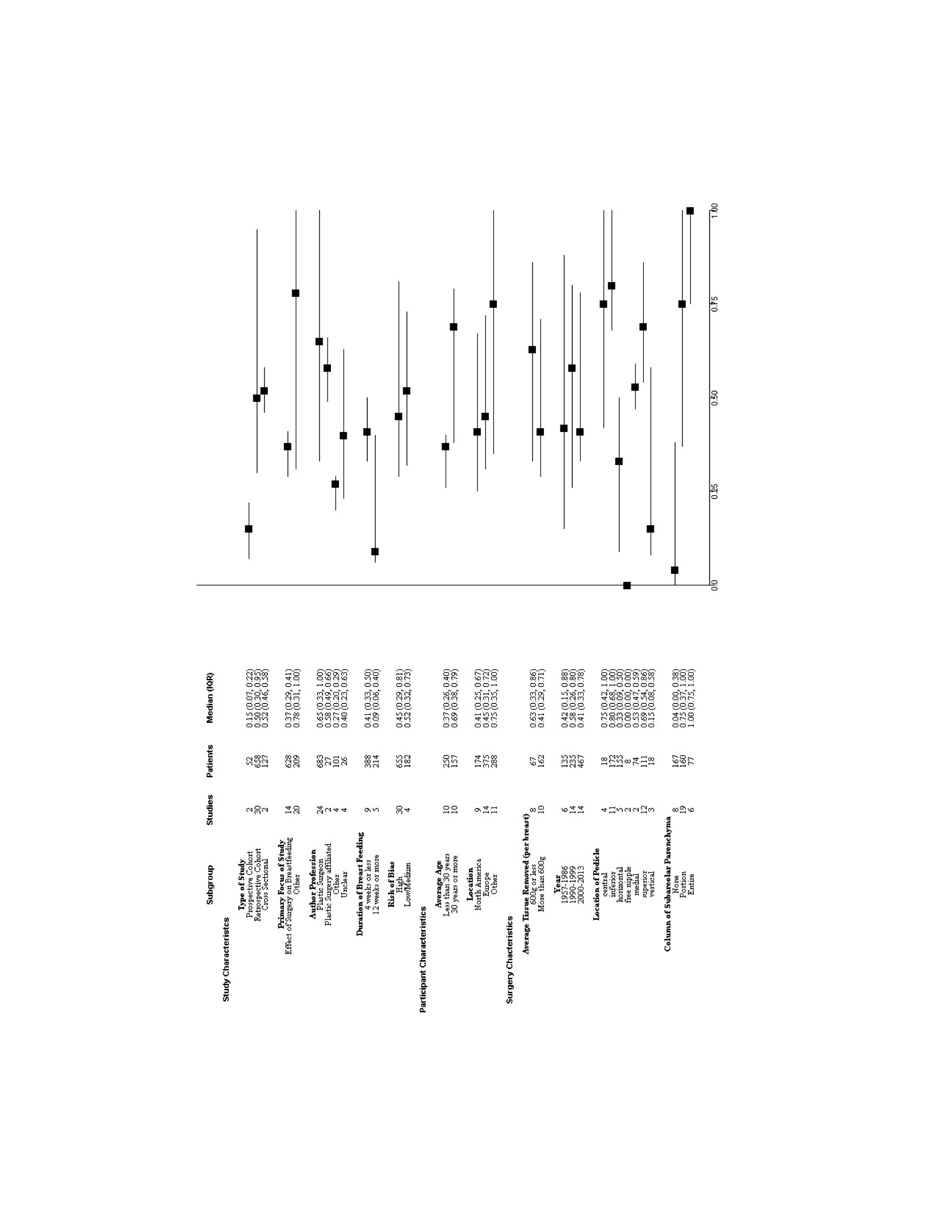
**
